# Their Desirability in General Hospitals

**Published:** 1914-04-25

**Authors:** 


### CLINICS FOR MENTAL DISEASE.

### Their Desirability in General Hospitals.

The *Modern Hospital*, an American magazine of imposing size, contains in its number for March several articles on the desirability of establishing clinics for mental disease in general hospitals. The suggestion is as excellent as it is obvious, and it is extraordinary that there are but two hospitals in London in which such a clinic exists, and in both cases the clinic is limited to out-patients.

### The Problem of Incipient Cases.

Patients, of the class that are treated in hospitals, who are manifestly insane, are sent to mental hospitals, and these institutions, which have been established and are maintained for the very purpose of caring for such patients will always receive, and ought always to receive, the bulk of them. There is, however, a large class of persons who suffer from mental disorder and yet are not insane. If such persons are well-to-do, or, at any rate, are able to pay the charges, they can be received in registered hospitals for the insane and in licensed houses as voluntary boarders should they so desire; and if they do not care to be so treated they can be treated while they are in their own homes or going about their work by specialists experienced in mental disease. For patients of the hospital class there is no such resource. Unless they are insane, and can be certified, they have no access to special treatment or advice. There has been for some time what can scarcely be described as an agitation, but there has been a desire on the part of those who administer public asylums that the provision of the law permitting of residence as a voluntary boarder should be extended so as to allow such residence in asylums as well as in registered hospitals and licensed houses. Such a provision would be an unmixed benefit, but it would still leave a large residue of persons suffering from mental disease without skilled treatment—practically without any treatment. There are many persons suffering from mental disorder whose disorder, while real enough and bad enough to poison their lives, is yet not bad enough to need residence in an institution of any kind; and for these, if they are of the social class from which hospital patients are drawn, there are no means of treatment. Such are the patients who now attend out-patient clinics for mental disease where such clinics exist.

### The Mental Hospital's Point of View.

The supply of treatment to those from whom it is now withheld would be but one, and perhaps not the greatest, benefit to be derived from clinics in mental disease. The cry of asylums has always been that they do not get their patients early enough. Persons, as a rule, do not go, or are not sent, to asylums until they are compelled to go by the severity of their malady, and by the time they are sent the early stage of their malady, and the stage most favourable for treatment, is already past. The out-patient clinic receives such patients in their earliest stage. There are no terrors in an out-patient clinic. No stigma attaches to the out-patient at a hospital such as attaches to an inmate of a mental institution. There is no more reluctance on the part of the patients to attend the department for mental diseases than to attend any other department.

### The Education of the Mental Physician.

But the benefits of the out-patient clinic are not confined to the patients. The physician for mental diseases sees in an out-patient clinic diseases and stages of disease that he never sees in an asylum. Indeed, it is scarcely too much to say that the education of the physician for mental diseases who has seen none but asylum cases is only half complete. He does not see the beginnings of insanity, and, therefore, is not on the alert to detect it in the early stages, and there are many forms of mental disease, not amounting to insanity, that he never sees at all. No asylum physician, unless he is also engaged in private practice, ever sees a case of imperative idea, or of obsession, or of claustrophobia, or of agoraphobia, or of the many other phobias and manias that do not attain to insanity. To the general physician such cases are of little interest, and he would be glad, when they present themselves in the general hospital, to shirk them off on to other shoulders, if there were any other department competent to take them. These minor mental diseases are entirely neglected. Their nature is so little known that they are usually confused with insanity, or thought to be of the nature of insanity, which they are not; and no treatment for them is known.

### The Law and In-Patient Clinics.

Out-patient clinics for mental diseases are manifestly most desirable, but there is no reason except lack of beds and lack of funds why there should not be in-patient clinics also. It is always assumed that such clinics would be illegal, and so they might be if they were to accommodate cases of insanity; but they could easily be filled without transgressing the law. It is a mistake to suppose that patients with mental disease would necessarily be a nuisance in a hospital, either to each other, to the rest of the patients, or to the administration; and, judging by other diseases, it may safely be predicted that our knowledge of the large field of mental diseases that do not amount to insanity will remain in its present elementary condition until hospital accommodation is provided for them.

